# Supplementary material for: Myoblast-Derived Galectin 3 Impairs the Early Phases of Osteogenesis Affecting Notch and Akt Activity
Source: Biomolecules. 2024 Sep 30;14(10):1243. doi: 10.3390/biom14101243 (PMC11505649; doi:10.3390/biom14101243)
Supplement: Supplementary file 1 [file biomolecules-14-01243-s001.zip › biomolecules-3137565-supplementary-figures and tables.pdf]

MYOBLAST-DERIVED GALECTIN 3 IMPAIRS THE EARLY PHASES OF OSTEOGENESIS AFFECTING NOTCH AND AKT ACTIVITY

SUPPLEMENTAL MATERIAL

Table S1: List of primers sequence used in this study.

**PRIMER**	**FW sequence**	**REV sequence**
GALECTIN 3	5'-AGCCAACGAGCGGAAAATGG-3'	5'-CCAGACCCAGATAACGCATCA-3'
HEY1	5’- TGTCTGAGCTGAGAAGGCTGGT-3’	5’- TTCAGGTGATCCACGGTCATCTG-3’
c-MYC	5’- GCGTTGGAAACCCCGACAG-3’	5’- GTCGTAGTCGAGGTCATAGTTCC-3’
GAPDH	5′-GGCATTGCTCTCAATGACAA-3′	5′-ATGTAGGCCATGAGGTCCAC-3′
HPRT	5′-CTCAGACCGCTTTTTGCCG-3′	5′-CATCATCGCTAATCACGACGC-3′

Table S2: List and concentration of use of antibodies used in this study.

ANTIBODY	DILUTION	COMPANY
Galectin 3	1:1000	Abcam Cambridge, UK
GAPDH	1:8000	Merck-Millipore, Darmstadt, Germany
FLAG	1:1000	Sigma-Aldrich, St. Louis, MO, USA
α/β-tubulin	1:2000	Sigma-Aldrich, St. Louis, MO, USA
ALP	1:1000	Santa Cruz, DBA Italia SRL, Segrate, Italy
NOTCH1	1:1000	Cell Signaling, MA, USA
MyoD	1:1000	Themo Scientific, Waltham, MA, USA
Secondary antibodies (HRP-conjugated)	1:12000	Abcam Cambridge, UK
Cleaved- NOTCH1	1:1000	Cell Signaling, MA, USA

Table S3: List of the proteins secreted by C2C12 cells differentiated with DMEM-HG plus 5 mg/ml insulin and identified by MASCOT. Proteins have been ordered based on the MASCOT protein score.

ID Uniprot	Protein name	Mass (kDa)	MASCOT Protein score
CO3A1_MOUSE	Collagen alpha-1(III) chain	140,112	1969
CO1A2_MOUSE	Collagen alpha-2(I) chain	129,992	1316
CO1A1_MOUSE	Collagen alpha-1(I) chain	138,974	501
LDHA_MOUSE	L-lactate dehydrogenase A chain	36,817	318
ANXA1_MOUSE	Annexin A1	38,995	307
ALDOA_MOUSE	Fructose-bisphosphate aldolase A	39,787	283
PGK1_MOUSE	Phosp hoglycerate kinase 1	44,921	192
ANXA2_MOUSE	Annexin A2	38,937	168
CO3A1_MOUSE	Collagen alpha-1(III) chain	140,112	144
CO1A1_MOUSE	Collagen alpha-1(I) chain	138,974	140
ACTB_MOUSE	Actin, cytoplasmic 1	42,052	140
FPPS_MOUSE	Farnesyl pyrophosphate synthase	40,898	132
CS1A_MOUSE	Complement C1s-A subcomponent	78,347	89
SFRP2_MOUSE	Secreted frizzled-related protein 2	34,473	79
SPB6_MOUSE	Serpin B6	42,913	73
LEG3_MOUSE	Galectin-3	27,612	64
PGS2_MOUSE	Decorin	40,126	61
SAHH_MOUSE	Adenosyl homocysteinase	48,17	59
CAPG_MOUSE	Macrophage-capping protein	39,501	59
CO5A1_MOUSE	Collagen alpha-1(V) chain	184,248	59
LMNA_MOUSE	Prelamin-A/C	74,478	58
TERA_MOUSE	Transitional endoplasmic reticulum ATPase	89,95	57
CATB_MOUSE	Cathepsin B	38,168	56
HS90B_MOUSE	Heat shock protein HSP 90-beta	83,571	55
EF2_MOUSE	Elongation factor 2	96,222	52
SPRC_MOUSE	SPARC	35,283	52
HS90A_MOUSE	Heat shock protein HSP 90-alpha	85,134	51
SODE_MOUSE	Extracellular superoxide dismutase [Cu-Zn]	27,717	50
AATC_MOUSE	Aspartate aminotransferase, cytoplasmic	46,504	46
MSLN_MOUSE	Mesothelin	70,177	46
ANXA3_MOUSE	Annexin A3	36,533	46
FINC_MOUSE	Fibronectin	276,017	45
ILEUA_MOUSE	Leukocyte elastase inhibitor A	42,719	45
NID2_MOUSE	Nidogen-2	156,61	44
AEBP1_MOUSE	Adipocyte enhancer-binding protein 1	129,026	43
1433E_MOUSE	14-3-3 protein epsilon	29,326	40
IDHC_MOUSE	Isocitrate dehydrogenase [NADP] cytoplasmic	47,044	39
ROA2_MOUSE	Heterogeneous nuclear ribonucleoproteins A2/B1	37,437	38
TIMP1_MOUSE	Metalloproteinase inhibitor 1	23,298	37
EF1A1_MOUSE	Elongation factor 1-alpha 1	50,424	36
CATL1_MOUSE	Cathepsin L1	38,093	36
ANXA5_MOUSE	Annexin A5	35,787	35
FSTL1_MOUSE	Follistatin-related protein 1	35,672	34
ROAA_MOUSE	Heterogeneous nuclear ribonucleoprotein A/B	30,926	33
MDHC_MOUSE	Malate dehydrogenase, cytoplasmic	36,659	29
TPM1_MOUSE	Tropomyosin alpha-1 chain	32,718	27
FINC_MOUSE	Fibronectin	276,017	27
TSP1_MOUSE	Thrombospondin-1	133,555	26
GELS_MOUSE	Gelsolin	86,287	24
MYH9_MOUSE	Myosin-9	227,429	22
IBP7_MOUSE	Insulin-like growth factor-binding protein 7	29,977	21
COPD_MOUSE	Coatomer subunit delta	57,649	21
DOC11_MOUSE	Dedicator of cytokinesis protein 11	240,242	21
MDHM_MOUSE	Malate dehydrogenase, mitochondrial	36,045	21
ACTB_MOUSE	Actin, cytoplasmic 1	42,052	20
ANXA4_MOUSE	Annexin A4	36,178	19
BIG3_MOUSE	Brefeldin A-inhibited guanine nucleotide-exchange protein 3	243,245	17
PHAG1_MOUSE	Phosphoprotein associated with glycosphingolipid-enriched microdomains 1	47,147	16

ID Uniprot	Protein name	Mass (kDa)	MASCOT Protein score
FINC_MOUSE	Fibronectin	276,017	652
VIME_MOUSE	Vimentin	53,712	552
PZP_MOUSE	Pregnancy zone protein	167,116	273
CO1A2_MOUSE	Collagen alpha-2(I) chain	129,992	188
A2MG_MOUSE	Alpha-2-macroglobulin-P	165,674	186
CO3A1_MOUSE	Collagen alpha-1(III) chain	140,112	182
CATB_MOUSE	Cathepsin B	38,168	128
CO4B_MOUSE	Complement C4-B	194,447	123
ALBU_MOUSE	Serum albumin	70,7	119
GELS_MOUSE	Gelsolin	86,287	105
CERU_MOUSE	Ceruloplasmin	121,872	102
PGS2_MOUSE	Decorin	40,126	77
PGS1_MOUSE	Biglycan	42069	75
PZP_MOUSE	Pregnancy zone protein	167,116	65
ACTB_MOUSE	Actin, cytoplasmic 1	42,052	64
FINC_MOUSE	Fibronectin	276,017	63
SPRC_MOUSE	SPARC	35,283	62
HPT_MOUSE	Haptoglobin	39,241	60
A2MG_MOUSE	Alpha-2-macroglobulin-P	165,674	58
EAF6_MOUSE	Chromatin modification-related protein MEAF6	21,636	55
NID1_MOUSE	Nidogen-1	139,302	51
TIMP2_MOUSE	Metalloproteinase inhibitor 2	24,996	49
CFAH_MOUSE	Complement factor H	143,722	48
MYPT2_MOUSE	Protein phosphatase 1 regulatory subunit 12B	109,326	47
NOS2_MOUSE	Nitric oxide synthase, inducible	131,861	46
MIME_MOUSE	Mimecan	34,333	44
FSTL1_MOUSE	Follistatin-related protein 1	35,672	41
TETN_MOUSE	Tetranectin	22,642	39
ATLA1_MOUSE	Atlastin-1	63,736	31
CYTC_MOUSE	Cystatin-C	15,749	29
PPIC_MOUSE	Peptidyl-prolyl cis-trans isomerase C	22,894	25

Mass spectrometric analysis of cell secretome (Supplemental information)

Coomassie stained bands were diced into 1 mm^3 cubes, destained for 30 minutes with 50% acetonitrile in 100 mM ammonium bicarbonate buffer. Samples were dehydrated with 100% acetonitrile for 10 minutes, reduced with 10 mM DTT (1,4-Dithiothreitol, Sigma Aldrich) for 30 minutes at 56°C and alkylated with 55 mM iodoacetamide (IAA, Sigma Aldrich) for 30 minutes in the dark. After alkylation, gel pieces were dehydrated with 100% acetonitrile and digested overnight with MS-grade trypsin (13 ng/µl in ammonium bicarbonate buffer 10nM with 10% acetonitrile; 90057, Pierce) at 37°C. The digestion products were extracted with a solution of 5% formic acid and acetonitrile 1:2 for 15 minutes at 37°C in a shaker and were then lyophilized using a SpeedVac Concentrator (Savant). Dry peptides from bands were re-suspended in 40 µL of a mixture of water: acetonitrile: formic acid 97:3:2, and separated on a Zorbax SB-C18 RRHT, 2.1x50mm, 1.8 µm particle size (Agilent Technologies) with a gradient from 3 to 21 acetonitrile in 19 minutes. The column was maintained at 30°C and the flow rate employed was 0.3 ml/min. The acquisition was performed in data-dependent acquisition mode and full MS scans were acquired in the Orbitrap. In each cycle, the top 5 most intense ions were selected for fragmentation. Fragment ion spectra were produced through collision-induced dissociation in a normalized collision energy of 28% and they were acquired in the ion trap mass analyzer. Raw data, converted into mascot generic format using MsConvert (v. 3.0.10730, ProteoWizard tools), were searched against Swiss-Prot (accessed June 2018; 16,977 sequences for Mus musculus) for peptide sequences and C-RAP for contaminants with MASCOT (Version 2.4, Matrix Science). Trypsin as proteolytic enzyme and carbamidomethyl cysteine as fixed modification were set in search parameters. Deamidated (NQ) and oxidated (M) were set as variable modifications. One missed cleavage was allowed. Mass tolerances were set at 10 ppm for the precursor ions and 0.05 Da for the product ions. Automatic decoy database search was used to estimate the false discovery rate, which was adjusted to 1%.

Identified proteins from C2C12 media are listed in Table 3S. Gal-3 and other previously reported myokines are highlighted.

.

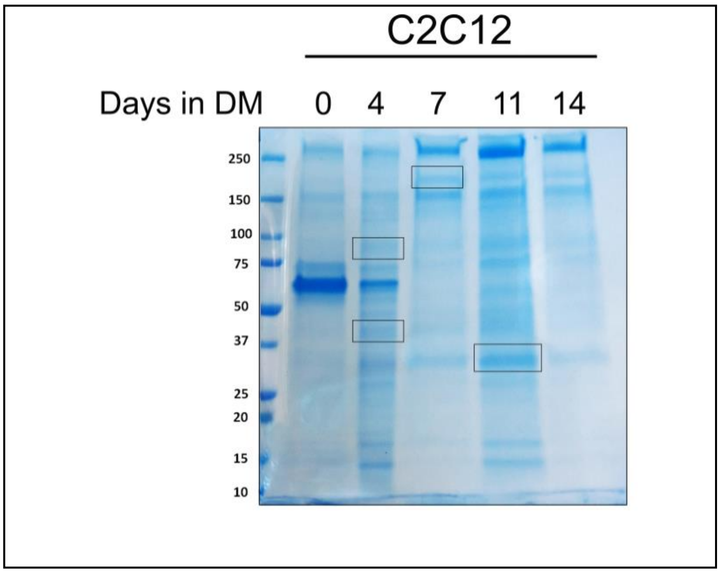

Figure S1: Mass spectrometric analysis of cell secretome.

Muscle-derived mouse C2C12 cells were differentiated in the presence of differentiation media (DM), consisting of DMEM-HG plus 5 mg/ml insulin. Culture media were collected after0, 4,7,11,14 days after medium change, and resolved in a gradient gel. Gel was next stained with Coomassie brilliant blue. Rectangular boxes represent enlarged portion of gel with the bands that were excised, pooled and analyzed by mass spectrometry. The bands inside the rectangular boxes have molecular weights in the range of 25 kDa to 37 kDa.

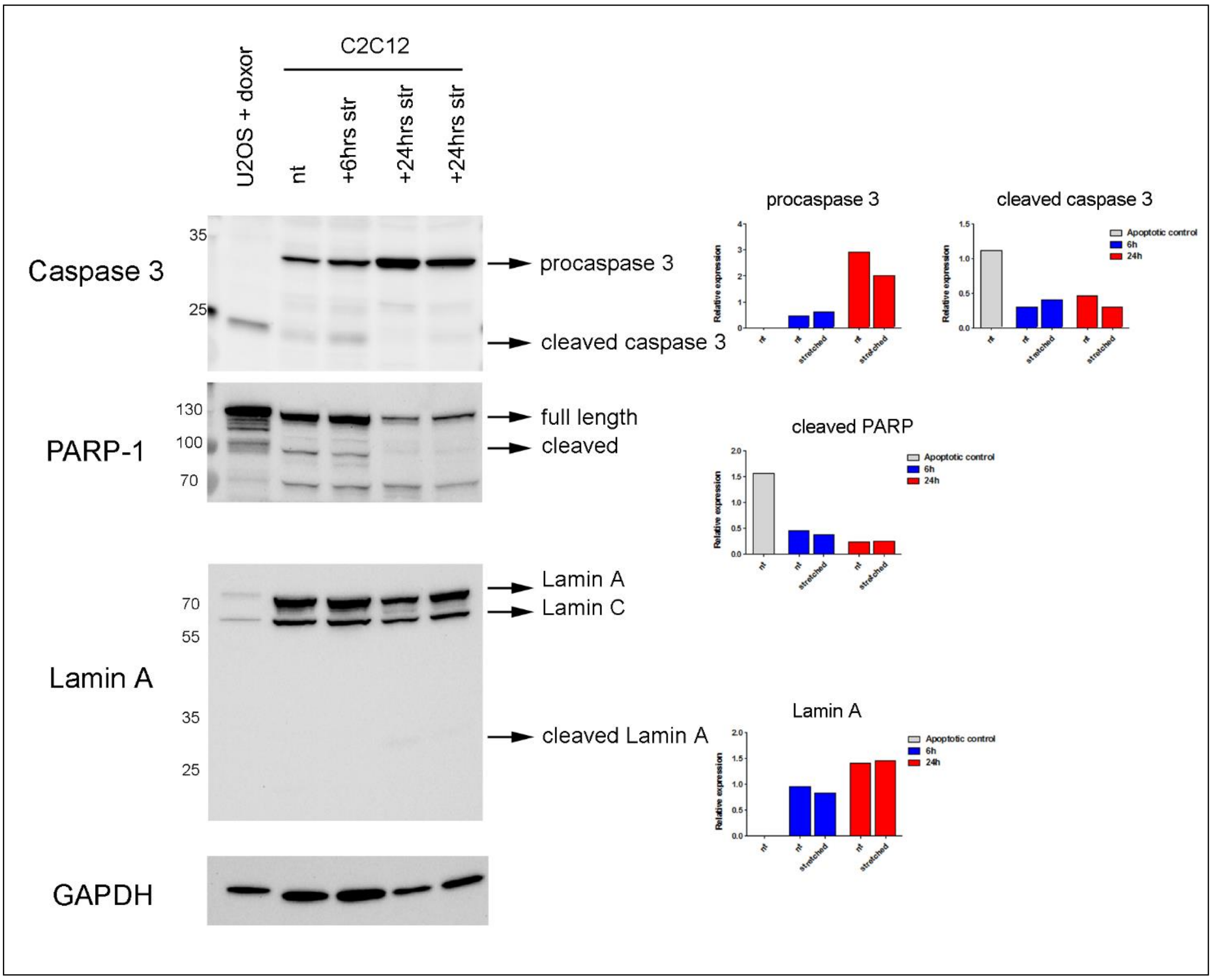

Figure S2: Apoptosis evaluation of C2C12 subjected to a stretching protocol.

Differentiated C2C12 were subjected to a multiaxial stretching (stretched) for 6 and 24 hours, or left untreated (nt). At the end of the stimuli, cells were lysed and together with corresponding media, subjected to immunoblot analysis to verify the presence of apoptotic cells by the use of specific markers: Caspase 3, PARP-1 and Lamin A. Bars are relative to two different experiments, * $p<0.05$. As apoptotic positive control an osteosarcoma cell line U20S was treated with Doxorubicin (0.5μM for 24 hours).

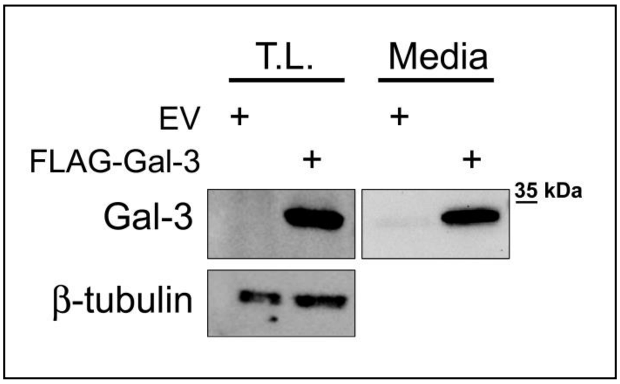

Figure S3: Level of expression and secretion of Gal-3 in C2C12 transfected with a plasmid encoding for murine FLAG-Gal-3.

C2C12 cells were transfected with a vector encoding for FLAG-tagged murine Gal-3 or with vector alone (EV). After 48 hours from transfection, cells were lysed and cultured media collected. Cell lysates (T.L.), together with corresponding media (Media) were resolved by SDS-PAGE and explored

for Gal-3. β-tubulin was used as equal loading control for total lysates.

Mass spectrometry analysis of cell lysates (Supplemental information)

After tryptic digestion, the peptide mixture was purified using Empore TM C18-SD 7mm/3ml extraction disk cartidge. Briefly, each column was activated by 100% acetonitrile (ACN) and stabilized by 70% ACN/0.1% formic acid (FA). Then, the samples were loaded into the column and centrifuged at 200xg for3 min. Peptides were washed with 0.1% TFA and subsequently eluted with 70% ACN. The eluted samples were dried up by Speed-vac. Peptides were resuspended in 2% ACN, 0.1% FA and separated on a 10 cm C18 Hypersil gold column (Thermo Scientific, Milan, IT) with a gradient from 2 to 28 acetonitrile in 90 min. The column was maintained at 30°C and the flow rate employed was 0.3 ml/min. The acquisition was performed in data-dependent acquisition mode and full MS scans were acquired in the Orbitrap. In each cycle, the top 7 most intense ions were selected for fragmentation. Fragment ion spectra were produced through collision-induced dissociation in a normalized collision energy of 28% and they were acquired in the ion trap mass analyzer. Raw ms/ms data were converted by msConvert ProteoWizard (v.3.0.19239) in .mgf file using default settings and uploaded to the MASCOT server (v.2.7.0) for MS/MS Ion Search. Search was performed using the SwissProt database (2022_02) restricted to Mus Musculus, and the reverse decoy database (cRAP) was added to calculate the false discovery rate (FDR) due to random match. Furthermore, parameters for identification included: (i) trypsin as enzyme with 1 of maximum missed cleavage; (ii) mass error tolerances for precursor and fragment ions, set to 10 ppm and 0.02 Da, respectively; (iii) peptide charge (2+, 3+, 4+) and (iv) carbamidomethyl cysteine (C), set as fixed modification, while deamidation of asparagine and glutamine (NQ) and oxidation of methionine (M) were considered as dynamic modification. The FDR for protein identification based on sequency homology was set to 1%. We performed label-free quantification using a tandem mass spectra counting approach (23), as already described (24, 25). Briefly, the exponentially modified protein abundance index (emPAI), integrated into Mascot, was calculated as the ratio between the number of experimentally observed peptides per protein and the number of theoretically observable peptides per protein. All emPAI values obtained were normalized, dividing each emPAI value for the sum of all emPAI values (26). The expression levels of the proteins identified were evaluated by fold changes. For each protein, fold change was obtained by dividing the emPAI average of (D3 + Gal-3) by that of (D3). If the p-value was < 0.05, an expression fold change greater than 4 was considered to indicate significant upregulation, whereas a fold change less than -4 was considered to indicate significant downregulation.

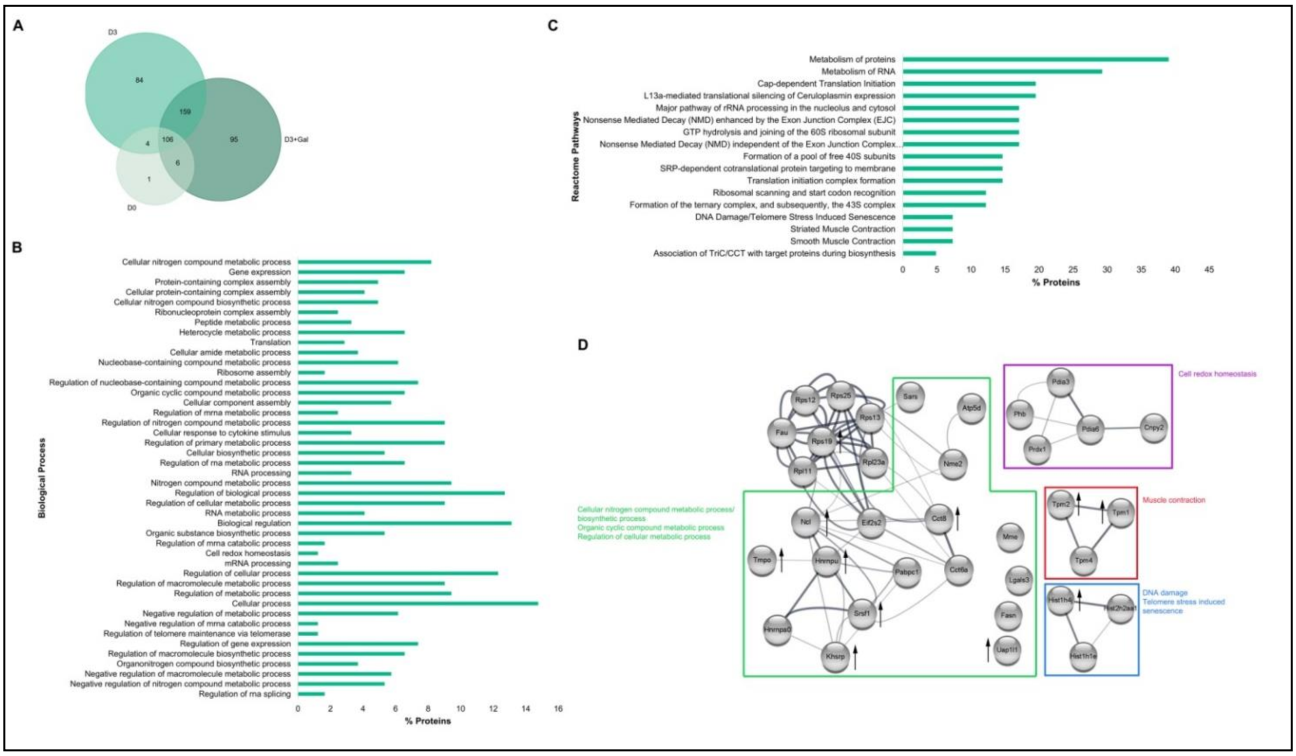

Figure S4: Venn diagram showing proteins identified by LC-MS analysis.

A) 117 proteins were identified in D0, 366 in D3 and 353 in D3 + Gal-3. Comparison between D0 and D3/D3 + Gal3 confirmed that proteins related to cell differentiation were dynamically expressed (see Supplementary Table S3). Therefore, we focused our analysis on the effect of Gal-3 during the differentiation. On the 264 proteins found to be expressed in both D3 and D3 + Gal-3, spectral counting analysis was performed: 12 proteins were considered significantly up-regulated and 1 protein down-regulated (Figure 3B). 84 and 95 proteins were uniquely identified in D3 and D3 + Gal3, respectively. **B-C)** Bars represent GO enrichment for biological process and Reactome Pathway for those proteins up/down regulated and uniquely identified in D3 + Gal-3. **D)** String network originated from D3 + Gal-3 proteins differentially expressed with respect of D3 (up or down-regulated) and uniquely identified (with at least 2 unique peptides). GO terms for biological process and the Reactome Pathway were interrogated showing that annotated protein complexes belong to cellular nitrogen compound metabolic process/biosynthetic process/organic cyclic compound metabolic process/regulation of cellular metabolic process (Pabpc1, Khsrp, Hnrnpa0, Nme2, Mme, Hnrnpu, Fasn, Eif2s2, Sars, Uap1l1, Atp5d, Rps19, Lgals3, Srsf1), cell redox homeostasis (Phb, Prdx1, Pdia3, Pdia6, Cpny2), muscle contraction (Tpm1, Tpm2, Tpm4) and DNA damage/telomere stress induced senescence (Hist1h1e, Hist1h4j, Hist2h2aa1). Arrows represent up/down regulated proteins resulted from spectral counting analysis.
