# Supplementary figures and images for: Myoblast-Derived Galectin 3 Impairs the Early Phases of Osteogenesis Affecting Notch and Akt Activity
Source: Biomolecules. 2024 Sep 30;14(10):1243. doi: 10.3390/biom14101243 (PMC11505649; doi:10.3390/biom14101243)

Fig 1C

Stretching            -   6h   -   24h

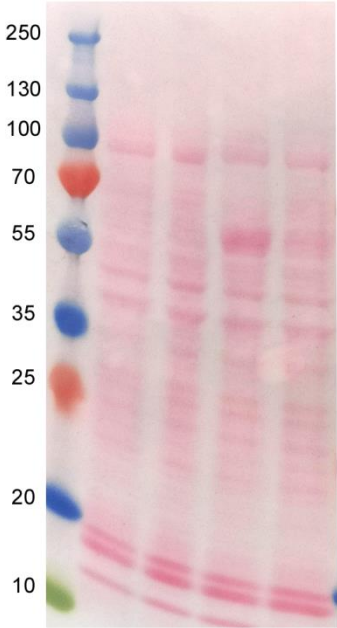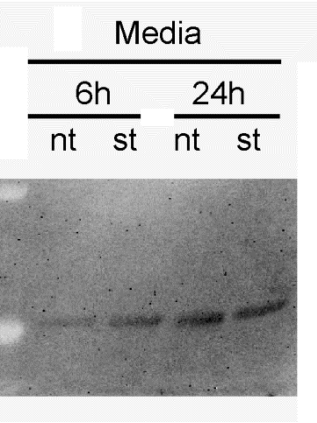

Galectin 3

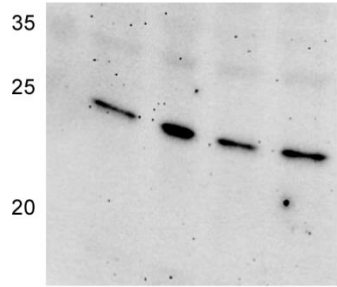

$\beta$ -Tubulin

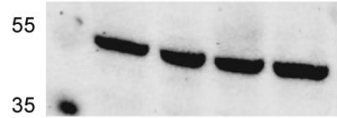

Fig 2 A

Gal 3

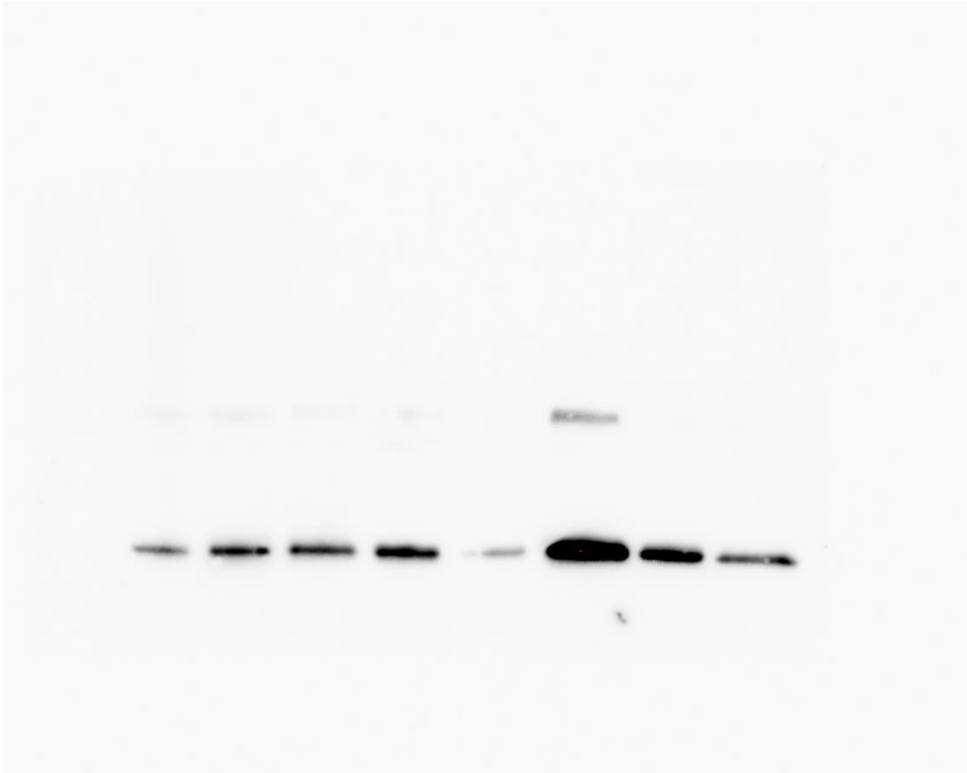

GAPDH

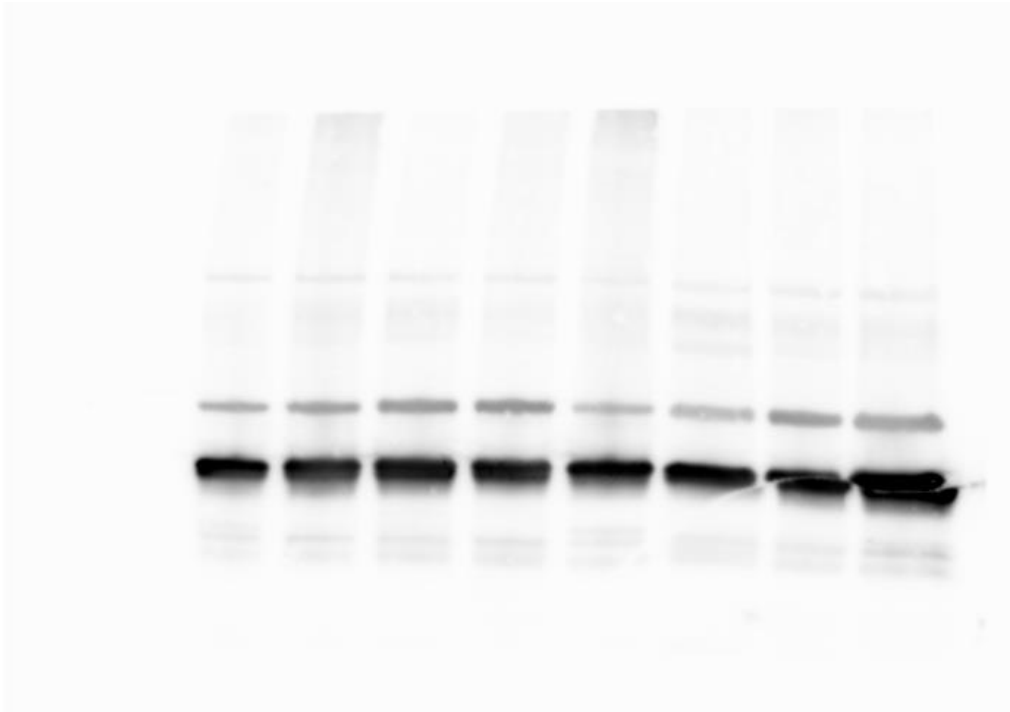

Fig 3 B

ALP

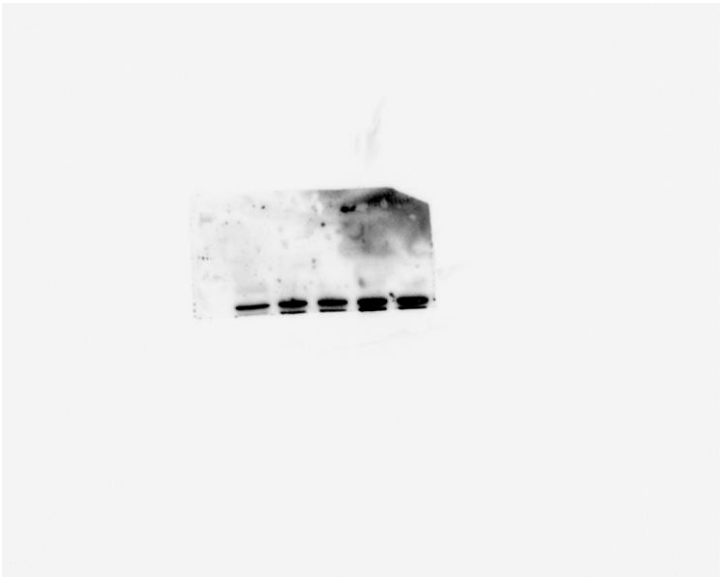

GAPDH

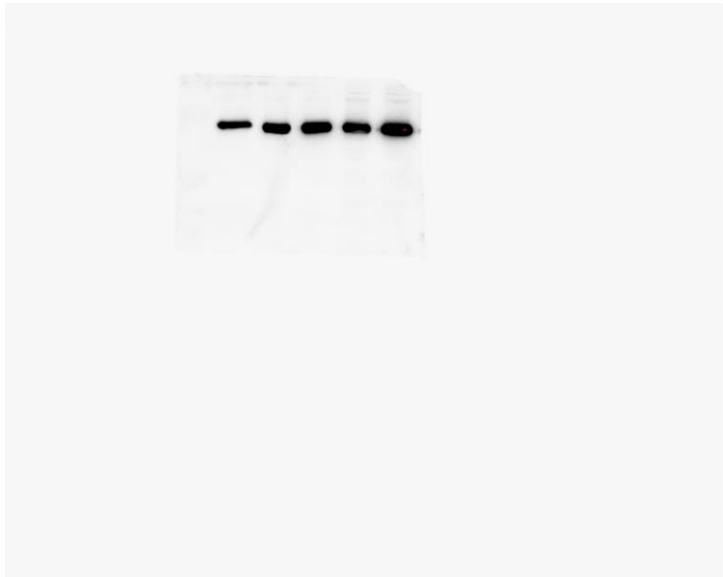

Fig 3 E

GAPDH

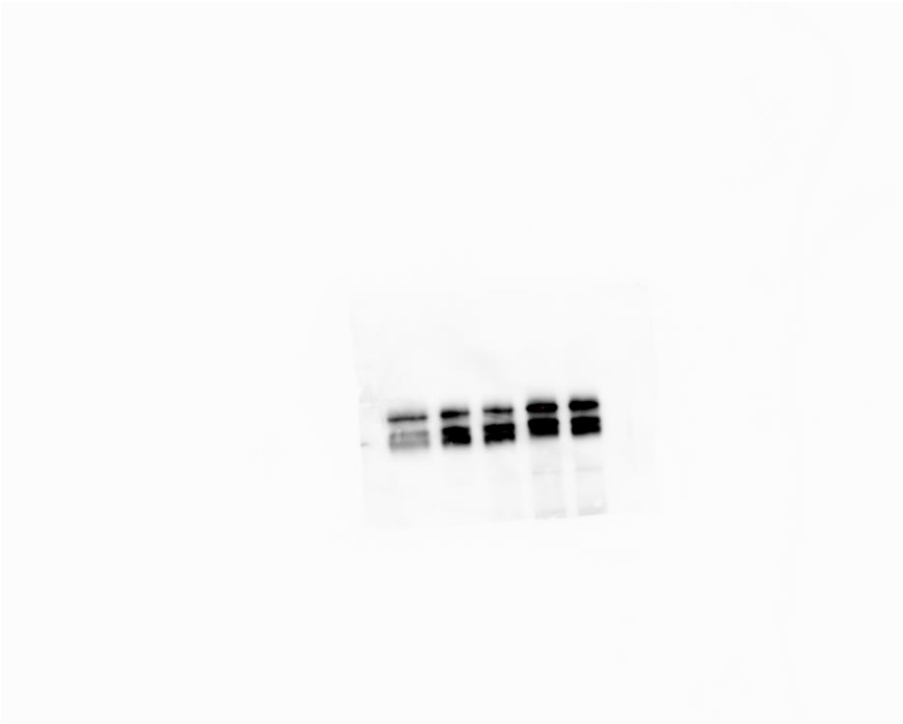

NOTCH1

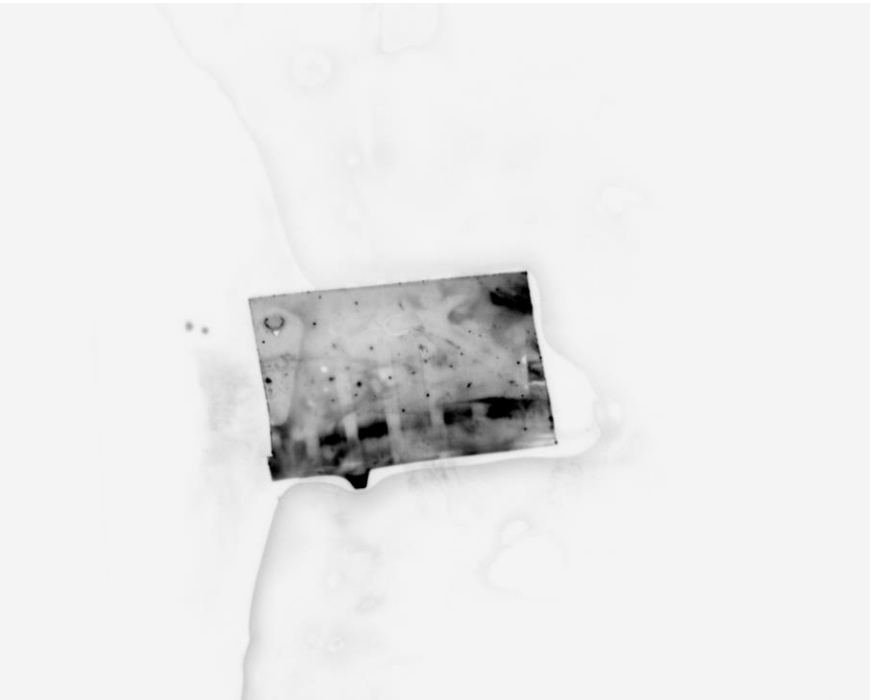

Supplement: Supplementary file 1 [file biomolecules-14-01243-s001.zip › biomolecules-3137565-supplementary-file S1.pdf]
